# Delayed Detection of a Carcinoid Tumor after Conservative Therapy for Appendicitis in a 13-Year-Old Boy and a 17-Year-Old Girl

**DOI:** 10.1055/s-0041-1728723

**Published:** 2021-11-10

**Authors:** Leonie Annina Korsch, Thomas Michael Boemers, Peter Zimmermann, Martin Stenzel, Wera Wendenburg

**Affiliations:** 1 Department of Pediatric Surgery and Urology, Kliniken der Stadt Köln gGmbH, Cologne, Germany; 2 Department of Pediatric Surgery, University of Leipzig, Leipzig, Germany; 3 Department of Pediatric Radiology, Kliniken der Stadt Köln gGmbH, Cologne, Germany

**Keywords:** acute appendicitis, conservative antibiotic treatment, carcinoid tumor, neuroendocrine tumor

## Abstract

Acute appendicitis is common in children and adolescents. Recently, conservative antibiotic treatment is regarded to be a safe approach to treat uncomplicated appendicitis. It is already established as initial treatment in cases of perforated appendicitis with perityphlitic abscess, commonly followed by interval appendectomy. We report on a 13-year-old boy with uncomplicated appendicitis and a 17-year-old girl with complicated, perforated appendicitis and perityphlitic abscess in whom initially successful antibiotic treatment led to a delay in detection of a carcinoid tumor (neuroendocrine tumor, NET) of the appendix. NET of the appendix, with an incidence of 0.03 to 0.8% in the pediatric population undergoing appendectomy for acute appendicitis, are usually incidental findings after appendectomy with no secure method for detection prior to surgery. Raising concern about this rare but severe disease, we recommend information of patients and their parents about the potential risk of belated diagnosis before opting for conservative their treatment of acute appendicitis. Furthermore, patients successfully treated conservatively require a close follow-up by ultrasound. In presence of any conspicuous finding, especially on imaging, appendectomy should be considered.

## Introduction

Acute appendicitis is common in children 1 2 and has a lifetime incidence of 7 to 14%. 3 Recently, conservative antibiotic treatment of acute appendicitis has been of growing interest 2 4 5 and reported to be successful in 62 to 95.5% of cases. 1 2 4 6

General guidelines for this treatment strategy, as well as long-term data of outcome in the pediatric population, are currently missing. 4 5 After successful antibiotic treatment of uncomplicated appendicitis, however, the potential risk of a delayed diagnosis of malignant processes of the appendix remains. 7

In contrast to the conservative treatment of uncomplicated appendicitis, the initial non-operative therapy with interval appendectomy for complicated appendicitis with appendiceal abscesses is well established and has advantages in terms of postoperative complications, especially regarding long-term obstruction events, and should be considered the first treatment of choice for pediatric patients with complicated appendicitis. 8 9

Carcinoid tumors of the appendix, which are highly differentiated NETs, are usually found incidentally in the course of an appendectomy due to acute appendicitis, with an incidence of 0.08 to 0.3% of children undergoing appendectomy. 10 11 12 Despite their rare occurrence, NETs of the appendix are a severe condition, raising concern, as to whether or not antibiotic treatment of acute appendicitis may result in diagnostic failure of this tumor. 7 13 We report on two cases in which nonoperative antibiotic treatment of an acute uncomplicated appendicitis and of a complicated appendicitis with periappendiceal abscess, respectively, led to a delay in the diagnosis of a NET of the appendix.

## Case 1

**Fig. 1:**
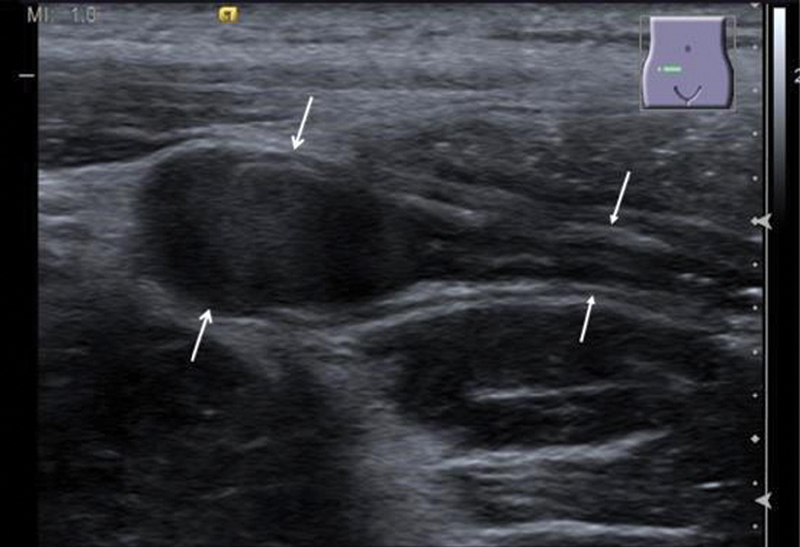
There is marked thickening of parts of the appendix up to a diameter of 7 mm (open arrowheads) compared with the normal adjacent part of the appendix (closed arrowheads). Technique: B mode sonography and color-coded sonography, Siemens ACUSON S2000, 14 MHz linear probe.

A 13-year-old boy presented with pain in the lower right abdomen, which had lasted for 2 days. He was otherwise healthy and without any comorbidity. He had no prior episodes of abdominal pain, nor a history of weight loss or intermittent fever. Symptoms of carcinoid syndrome were absent as well. On clinical examination, he showed the classical signs of an acute appendicitis. His temperature was 36.5°C (97.7°F). Initial inflammatory parameters were within normal range with leukocytes of 5.1/nL, neutrophils of 2.6/nL (51.3%), and a C-reactive protein (CRP) of <3 mg/L. On ultrasound, the appendix was displayed over a long distance with a maximum caliber of 7 mm at the apex surrounded by a slight layer of free fluid showing the ultrasonographic picture of an acute appendicitis. An appendicolith was not present. Due to the mild clinical presentation, as well as the boy's and his parents' preferences, we opted for antibiotic treatment, which soon led to complete resolution of symptoms. An ultrasound 2 days later showed a reduction of the appendix size (maximum 5 mm). The patient was released after 3 days of intravenous antibiotic treatment (ampicillin/sulbactam) and oral antibiotic application was continued for 10 days. In want of general guidelines for monitoring therapeutic success of conservative treatment of acute appendicitis, we routinely perform an ultrasound 6 weeks after patient's discharge for follow-up. In this examination, the apex of the appendix of the boy was distended to a diameter of 7 mm, showing hyperemia and missing visualization of bowel layers as well as surrounding perityphlitic edema ( Figs. 1 2 3 ), which lead to the suspicion of chronic appendicitis. At this time, the patient was free of symptoms and inflammatory parameters were within normal ranges again (leukocytes 5.0/nL, neutrophils 2.3/nL [46.9%], and CRP < 3 mg/L). An appendectomy was performed using single-port technique with the finding of a phlegmonous appendicitis without other abnormalities. Histology revealed a 1.4 cm measuring G2-NET at the apex of the appendix. The tumor was classified according to Union international contre le cancer (UICC) as pT4, pNX, L0, V0, and Pn0 with tumor cells present at the outer surface of the serosa. Resection margins were free of tumor cells. Chromogranin A and 5-hydroxyindoleacetic acid were within normal range and magnetic resonance imaging (MRI) of the abdomen revealed no suspicious lymph nodes. The patient has been well for 3 months since discharge in periodic health examinations up to date.

**Fig. 2:**
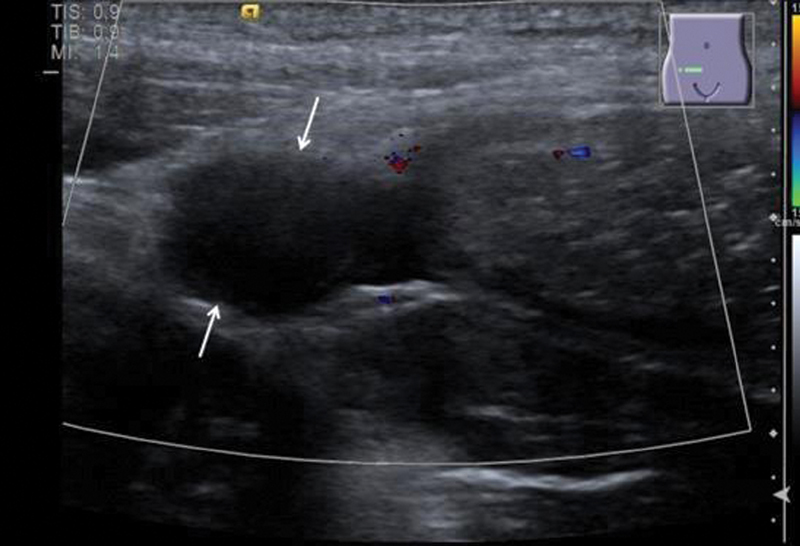
The thickened appendix shows complete loss of the regular bowel layers, in general is hypoechoic (between arrows). There is no marked hyperperfusion of the tissue.

**Fig. 3:**
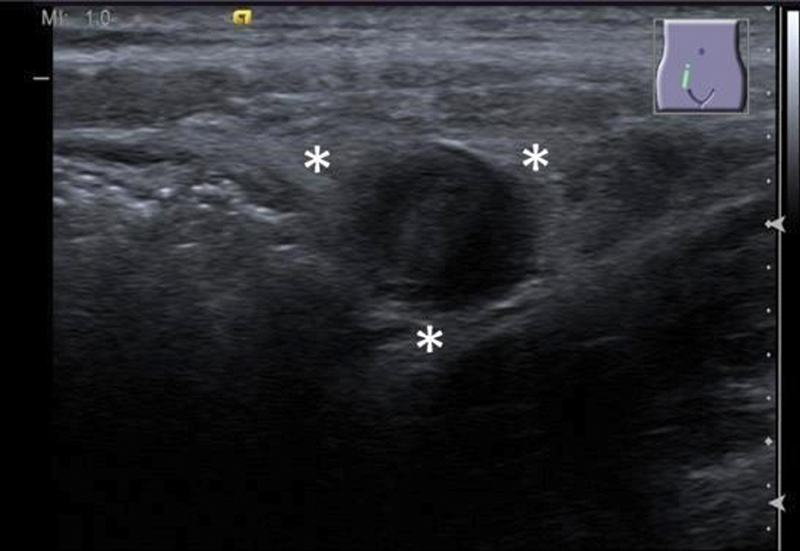
The periappendiceal tissue is hyperechoic, representing edema (asterisks).

## Case 2

A 17-year-old girl was transferred to us from a primary care hospital with suspected perityphlitic abscess. She was presented there 2 days prior with pain in the lower right abdomen for 5 days, diarrhea, and fever up to 39.6°C (103.3°F) as well as elevated CRP (134 mg/L). Her gynecologic history was empty. Abdominal imaging (abdominal ultrasound and CT) showed a perityphlitic abscess and the patient was transferred to our tertiary center. On clinical examination, she presented with moderate pressure pain in the right lower abdomen, without rebound tenderness, percussion pain, or guarding. She had a normal heart rate, no fever, and her CRP was 200 mg/L. An intravenous hydration as well as an antibiotic therapy with imipenem was initiated. An additional MRI of the abdomen showed a multichamber abscess with the right ovary included into the inflammatory mass. The abscess was drained by ultrasound-guided transrectal puncture under general anesthesia. Microbiology displayed imipenem-sensitive *Escherichia coli* and *Candida albicans* , wherefore an antimycotic intravenous therapy with caspofungin was added. Due to persistent abdominal distention and paralysis but without peritonitis as well as an increase in inflammatory parameters (CRP up to 290 mg/L and leukocytosis with 39.1/nL), another MRI was performed. Two abscesses in the pouch of Douglas and dorsolateral to the cecum were detected, which were treated with two drains placed under CT guidance by an interventional radiology. The further recovery of the patient was slow but uneventful. After 2 weeks, the drains were removed. Antibiotic and antimycotic therapies were stopped 1 week later. The girl was discharged in good condition, with normal laboratory findings and full enteral feeds 4 weeks after admission. One month later, we performed an elective appendectomy in single-incision laparoscopic surgery technique. Intraoperatively, there were postinflammatory adhesions in the right lower abdomen, hyperemia, edema, and moderate fibrinous exudate of the appendix. Histology revealed a well-differentiated NET measuring 1.4 cm in largest diameter with infiltration into the mesenteriolum (infiltration depth: 0.2 cm), and no infiltration of lymphatic vessels, blood vessels, or perineural sheaths. The resection margins were free of tumor cells resulting in a grading: G1; pT3, pNX M0, L0, V0, and Pn0 (UICC stage II). The levels of chromogranin A and 5-hydroxyindoleacetic acid were within normal range. Since the tumor was smaller than 15 mm in diameter and the resection was complete, there was no indication for right hemicolectomy. The patient was included in the German Society for Paediatric Oncology and Haematology—Malignant Endocrine Tumours registry with 3-month follow-up intervals (abdominal ultrasound; tumor markers: chromogranin A and 5-hydroxyindoleacetic acid).

## Discussion

Watanabe et al were the first to report an adult, who was successfully treated conservatively for on acute appendicitis and showed the signs of acute appendicitis with a dilated lumen at the apex on a follow-up ultrasound 3 months later again. 14 Histology after appendectomy revealed a G1-NET of the appendix. 14 Gorter et al 7 questioned, whether conservative treatment of acute appendicitis in a patient with NET of the appendix would have led to clinical improvement. The examples of our patients, as well as the one presented by Watanabe et al, 14 show that temporary clinical resolution of symptoms, despite a NET of the appendix, is possible under nonoperative, antibiotic treatment in case of acute uncomplicated and complicated appendicitis with periappendiceal mass. In the case presented by Watanabe et al 14 as well as in Case 1, the recurrence of the acute appendicitis was detected on routine follow-up without causing any clinical symptoms. Without any follow-up or mandatory interval appendectomy, it is possible that our patients would have become symptomatic again, however, allowing for further tumor growth in the meantime, which might have made bowel resection mandatory. The NET of the appendix, in both cases presented, had a size of 1.4 cm. Compared with a cohort of nine patients treated for NET of the appendix in Department of Pediatric Surgery and Urology of Cologne's Children Hospital within 16 years, there are only two patients with an equal or larger tumor size. 15 Apart from acute appendicitis, NET of the appendix in childhood are clinically silent. 11 13 Reliable diagnostic measure for detection prior to surgery is missing, 12 especially as approximately 80% of NET of the appendix in histologic findings have a size of ≤1 cm 11 making them difficult to visualize on ultrasound and even on MRI or CT. 14 Therefore, Watanabe et al 14 suggested considering routine interval appendectomy following conservative treatment of acute appendicitis to detect possible NET, especially if distension of the distal lumen of the appendix remains present on ultrasound. If in conclusion conservative treatment of appendicitis, whether uncomplicated or complicated, should however generally require interval appendectomy to prevent potential overlook of malignancy, it will raise the question of benefit for primary antibiotic treatment in the treatment of uncomplicated appendicitis, which in particular is to avoid surgery 3 4 7 with the associated risks. 1 Requiring two hospital stays, patients' morbidity would thereby be increased compared with appendectomy as first-line procedure.

Nevertheless, for complicated appendicitis with periappendiceal abscess, initial antibiotic therapy followed by interval appendectomy has advantages in terms of a lower complication rate, including surgical wound infection, postoperative peritonitis, or formation of a reabscess as well as lower incidence of adhesive small bowel obstruction.

If antibiotic treatment of acute appendicitis is primarily chosen, clinics need to be aware of the potential risk of NET of the appendix being missed, however, rare this tumor entity may be. This emphasizes out on the necessity of follow-up imaging after successful conservative treatment of acute appendicitis. The examples of patients given in this article underline that radiological signs of appendicitis may be visible on imaging, even in a patient free of symptoms. Though a NET is not likely to be detected, it may provoke anatomic a aberrations of the appendix. In our opinion, any abnormality should lead to perform appendectomy. In this course, in clinic or imaging appendectomy should be generally suggested for recurrent appendicitis following conservative treatment of acute appendicitis not to miss a hidden NET.

Furthermore, in the case of a conservative treatment option, the possible risk of delayed detection of NET of the appendix 13 should be part of the initial therapy enlightenment 3 , as a missed diagnosis of NET may lead to a more advanced stage of the disease 12 on detection, potentially requiring an invasive therapy such as follow-up bowel resection. An appropriate enlightenment of tumor risk is necessary for adequate empowerment of the patient's and the parents' decision-making. 3

## Conclusion

After nonoperative, antibiotic treatment of appendicitis, follow-up ultrasound is required. An interval appendectomy should be performed in any case of conspicuities to reveal a hidden carcinoid tumor (NET), according to the medical dogma “if in doubt take it out.” Furthermore, the risk of delayed detection of a possible NET due to conservative therapy needs to be part of the initial patient's and parents' enlightenment for this option of therapy, especially in case of uncomplicated acute appendicitis.

## Notes

New Insights and the Importance for the Pediatric Surgeon We report on two cases, a 13-year-old boy and a 17-year-old girl with a carcinoid tumor (neuroendocrine tumor, NET) of the appendix, in whom initial conservative therapy of an otherwise uncomplicated acute appendicitis (13-year-old boy) and complicated perforated appendicitis with abscess (17-year-old girl) led to a delay in detection and treatment of appendiceal NET.
